# Endovascular treatment of an aneurysm associated with fenestration of the supraclinoid internal carotid artery: Case report and review of the literature

**DOI:** 10.3389/fneur.2022.966642

**Published:** 2022-11-09

**Authors:** Rareṣ Cristian Filep, Cristian Constantin, Emil Marian Arbǎnaṣi, Adrian Vasile Mureṣan, Eliza Russu, Lucian Mǎrginean

**Affiliations:** ^1^Department of Clinical and Medicosurgical Disciplines - Radiology, George Emil Palade University of Medicine, Pharmacy, Science, and Technology of Targu Mures, Targu Mures, Romania; ^2^Department of Radiology and Medical Imaging, University of Medicine and Pharmacy of Craiova, Craiova, Romania; ^3^Clinic of Vascular Surgery, Mures County Emergency Hospital, Targu Mures, Romania; ^4^Department of Surgery, George Emil Palade University of Medicine, Pharmacy, Science, and Technology of Targu Mures, Targu Mures, Romania

**Keywords:** fenestration, intracranial aneurysms, endovascular, neuroradiology, internal carotid artery

## Abstract

**Background:**

Fenestrations or divisions of the vascular lumen into separate channels appear to be common anatomical variations in patients with intracranial aneurysms. The most frequent sites of occurrence are the anterior communicating artery (ACom), followed by vertebrobasilar and middle cerebral artery (MCA) locations.

**Case presentation:**

A 61-year-old female was brought to the emergency department after experiencing severe headache with abrupt onset, nausea, and vomiting. Clinical examination on arrival showed a drowsy patient (GCS 14), with neck stiffness, but no cranial nerve palsies or other neurological deficits (Hunt-Hess 2). Non-contrast head CT and CT angiography revealed subarachnoid and intraventricular hemorrhage (modified Fisher 4) and two saccular aneurysms, one located on the right supraclinoid ICA with peripheral calcifications, measuring 20 × 12 mm, the second on the left MCA bifurcation, 6 × 4 mm. 3D rotational angiography revealed a right ICA fenestration located between the ophthalmic (OA) and posterior communicating artery (PCom). The proximal part of the fenestration harbored a large saccular aneurysm projecting superiorly with the neck engulfing the origin of the fenestration; due to the favorable neck and geometry of the aneurysm, endovascular coil occlusion was chosen as a treatment option without balloon or stent assistance. The decision was taken to clip the MCA aneurysm.

**Conclusion:**

Supraclinoid ICA fenestrations are rare anatomical variations. Endovascular treatment of supraclinoid ICA fenestration-related aneurysms is feasible and safe, with the notable concern of perforators originating from the limbs.

## Introduction

Fenestrations or divisions of the vascular lumen into separate channels appear to be common anatomical variations in patients suspected with intracranial aneurysms. The most frequent sites of occurrence are: the anterior communicating artery (ACom), followed by vertebrobasilar and middle cerebral artery (MCA) locations (1). The internal carotid artery (ICA) is an extremely rare location for this anomaly, only 23 cases being reported in the medical literature so far.

We present the case of an acutely ruptured saccular aneurysm developed on the proximal part of a supraclinoid ICA fenestration, treated by endovascular coil embolization; a review of the literature regarding supraclinoid ICA fenestrations was also conducted.

## Case report

A 61-year-old female was brought to the emergency department after experiencing severe headache with abrupt onset, nausea, and vomiting. Clinical examination on arrival showed a drowsy patient (GCS 14), with neck stiffness, but no cranial nerve palsies or other neurological deficits (Hunt-Hess 2); her blood pressure was 190/110 mmHg. Other laboratory blood tests were within normal ranges.

**Figure 1:**
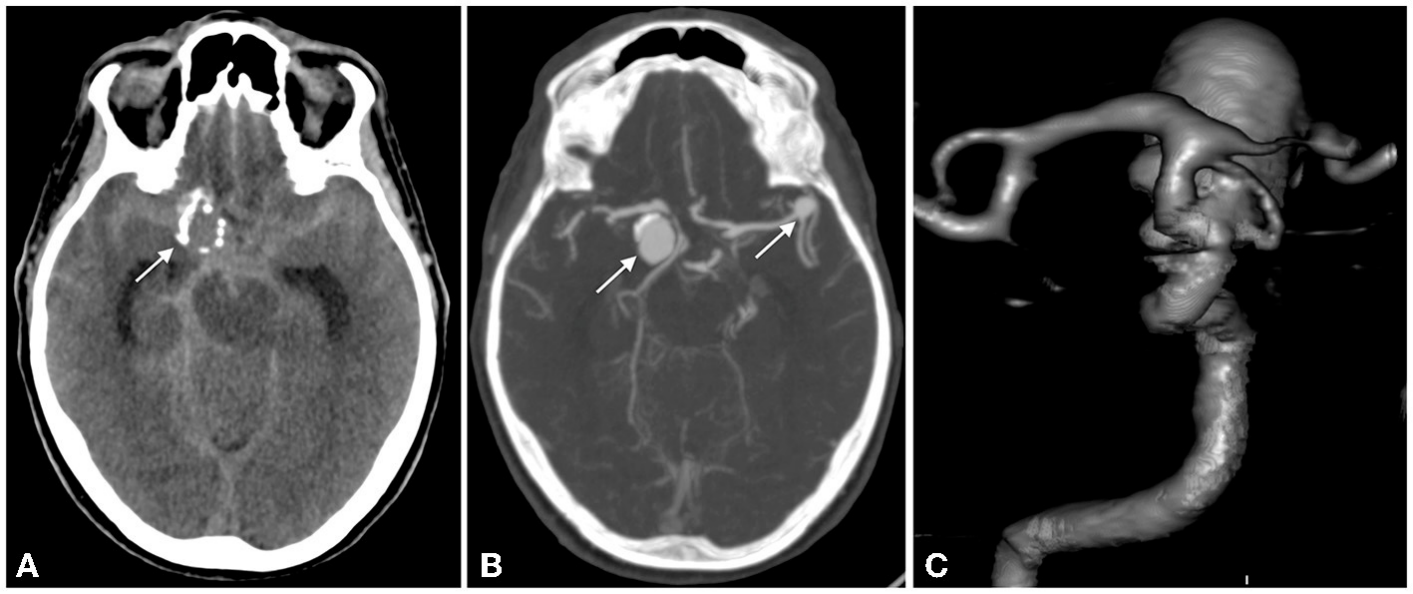
NCCT: subarachnoid hemorrhage in the basal and sylvian cistern >1 mm thick and in the 4^th^ ventricle; a round mass with peripheral calcification is seen (white arrow) **(A)**; CTA and VRT display 2 aneurysms, one on the right supraclinoid ICA, the second on the left MCA bifurcation (white arrows) **(B,C)**.

Non-contrast head CT and CT angiography revealed subarachnoid and intraventricular hemorrhage (modified Fisher 4) and two saccular aneurysms, one located on the right supraclinoid ICA with peripheral calcifications, measuring 20 × 12 mm, the second on the left MCA bifurcation, 6 × 4 mm (Figure 1). Due to its displacement by the aneurysm, only retrospectively was the fenestration identified on the CTA (not shown). Due to a larger maximal diameter, a higher aspect ratio, and the presence of a bleb, the ICA aneurysm was considered the most likely culprit of the subarachnoid hemorrhage.

**Figure 2:**
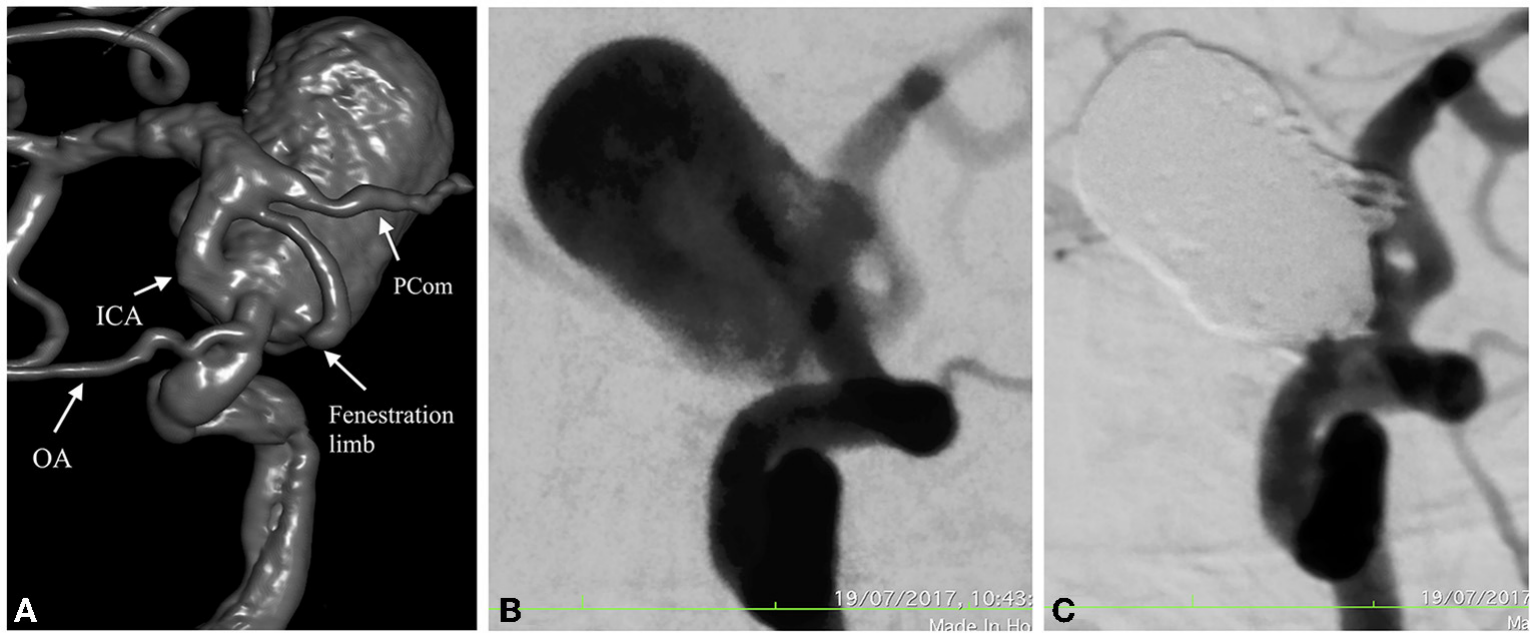
3D Angio in oblique views show the fenestration arising distal to the OA and ending proximal to a fetal type PCom **(A)**; DSA in working projection depicts the aneurysm before and after endovascular coil embolization with near complete obliteration of the sac (Raymond-Roy Occlusion Classification IIIb) **(B,C)**.

Catheter angiography was performed the next day on a biplane Siemens Artis Zee machine (Siemens, Erlangen, Germany); 3D rotational angiography revealed a right ICA fenestration located between the ophthalmic (OA) and posterior communicating artery (PCom). The proximal part of the fenestration harbored a large saccular aneurysm projecting superiorly with the neck engulfing the origin of the fenestration (Figure 2A); due to the favorable neck and geometry of the aneurysm, endovascular coil occlusion was chosen as a treatment option without balloon or stent assistance. The decision was taken to clip the MCA aneurysm at a later stage because of its wide neck and the necessity to perform stent-assisted coiling, requiring dual antiplatelet therapy.

With the patient under general anesthesia, a right femoral approach *via* a 6F sheath (Cordis Corporation, California, USA) was used to navigate a 6F Chaperon guiding-catheter (Microvention, Tustin, California) in the distal part of the cervical ICA, through which a straight-tipped PxSlim (Penumbra, Alameda, California) microcatheter was introduced into the aneurysm sac, followed by packing with 8 Penumbra 400 coils (Penumbra, Alameda, California) until almost complete obliteration was achieved assessed by Raymond-Roy occlusion classification as IIIb (Figures 2B,C). All catheters were continuously flushed with saline and unfractioned heparin throughout the procedure. After introduction of the first coil, a bolus of 5,000 IU of unfractioned heparin was administered intravenously to prevent thromboembolic complications. An Angio-Seal (Terumo, Tokyo, Japan) closure device was used for hemostasis.

There were no intraprocedural complications. The patient received 10 ml/h i.v. nimodipine during hospitalization and her clinical condition and neurological status improved in the following days. Follow-up CT at 24 h did not show additional complications. The patient was discharged 15 days later with mRS 1, with a slight disorientation.

## Methods and materials

A MEDLINE search (PubMed) was performed, using the following terms: “Internal Carotid Artery” (all fields) AND “Fenestration” (all fields) sorted by “Best match”. The search generated 145 results. The inclusion criteria were imaging and/or surgical confirmation of a fenestration located on the ICA segments distal to the anterior clinoid process or ophthalmic artery. The authors independently read the titles, abstract, full text and relevant references. Out of the 145 results, 21 met the inclusion criteria and no relevant study was found in the references.

The study was conducted in accordance with the Declaration of Helsinki and approved by the Ethics Committee of Târgu-Mureṣ Emergency County Hospital, Romania (Protocol Code 30418, on December 7, 2021) and written informed consent has been obtained from the patient to publish this paper.

## Results

A total of 23 cases with supraclinoid ICA fenestration were published between 1983 and 2018 in the 21 articles which met the inclusion criteria (2–22); in all of them the fenestration began at or above the OA, and 18 had at least one associated aneurysm at the proximal and/or distal part (2, 6, 8, 10–17, 19–22). Only 3 out of the 23 cases had no intracranial aneurysms (4, 9, 18). Endovascular treatment was undertaken in 8 aneurysms (8, 14–16, 20–22), the rest being treated surgically. All cases are included in Table 1.

**Table 1:** Literature review of all published cases with supraclinoid ICA fenestrations.

**References**	**Age (years)**	**Sex**	**SAH**	**Associated aneurysm**	**Treatment**	**Aneurysms in other location**
Yock (2)	41	F	Yes	Yes	Wrapping	No
Findlay et al. (3)	28	F	Yes	No	No	ACom
Takano et al. (4)	51	F	No	No	No	No
Hattori and Kobayashi (5)	38	F	Yes	No	No	ICA bifurcation
Katsuta et al. (7)	46	F	Yes	No	No	SCA
Banach and Flamm (6)	37	F	Yes	Yes	Clipping	PCom
Ng et al. (8)	34	F	No	Yes, 2	Clipping/coiling	No
Bharatha et al. (9)	73	M	No	No	No	No
Chen et al. (10)	31	M	Yes	Yes	Wrapping	No
Onoda et al. (11)	42	F	No	Yes	Wrapping	n/a
Plumb et al. (12)	48	F	Yes	Yes	Clipping	No
Ichikawa et al. (14)	47	F	No	Yes	Coiling	ACho
Dey and Awad (13)	39	F	No	Yes	Clipping	n/a
	32	F	Yes	Yes	Clipping	n/a
Nakiri et al. (16)	47	F	No	Yes	Coiling	n/a
	44	M	Yes	Yes	Coiling	n/a
Park and Lee (15)	44	M	Yes	Yes	Coiling	MCA
Rennert et al. (17)	52	F	No	Yes	Clipping	ACho
Orru et al. (18)	26	F	No	No	No	No
Uchino and Tanaka (19)	73	F	No	Yes	No	No
Lee et al. (20)	65	F	No	Yes	Coiling	No
Sgreccia et al. (21)	55	M	No	Yes	Coiling + FD	MCA
Jha et al. (22)	60	F	No	Yes	Coiling + FD	No

SAH, subarachnoid hemorrhage; ACom, anterior communicating artery; ICA, internal carotid artery; SCA, superior cerebellar artery; PCom, posterior communicating artery; ACho, anterior choroidal artery; MCA, middle cerebral artery.

## Discussion

A fenestration is defined as a division of the vascular lumen in two separate channels, each with its own tunica intima, media and adventitia, although the adventitia can be shared (23). In a recent prospective angiographic study, intracranial artery fenestrations were found in 24% of patients with ruptured aneurysms. The ACom was the most frequent site of occurrence (69%), followed by the A1 segment of the anterior cerebral artery (ACA) (9%), MCA (9%), basilar artery (9%) and the vertebral artery (2%) (1). Intracranial ICA segments seem to be extremely rare locations affected by fenestrations, to our knowledge only 23 cases being reported in the literature so far (2–22).

The primitive ICA develops from the dorsal aorta during the first 3 stages of embryologic development [4–12 mm crown-rump length (CRL)] (6). In the first stage (4–5 mm CRL), both ICA's are connected by a network of plexiform anastomoses, which fuse to form the main ICA channels; it is during this stage also that the primitive ICA divides distally into a posterior trunk, the precursor of the PCom and an anterior trunk, the ACA with its branches (anterior choroidal artery, MCA); the division point is just distal to the ophthalmic artery.

Although not clearly understood, two main theories emerged as possible explanations for the development of supraclinoid ICA fenestrations:

- *Bifurcation failure* of the ICA into the anterior and posterior trunks;
- *Fusion failure* of the plexiform anastomoses between the two ICA's.

In our opinion, the second theory is more plausible because of the numerous reports of ICA fenestrations occurring in all segments, including cervical, petrous and cavernous (24, 25) suggesting that the ICA develops through fusion of numerous plexiform channels. An additional argument supporting the fusion failure theory is that intracranial vessels like the ACA, MCA and the vertebrobasilar system also develop through fusion of numerous vascular channels.

The association between fenestrations and aneurysms is not clearly understood. In their prospective angiographic study, van Rooij et al. (1) didn't find a significant relationship between them; on the contrary, out of the 24 cases with supraclinoid ICA fenestrations, including our own, 17 (74%) harbored one or more aneurysms at the fenestration site (Table 1) suggesting that a causative association is plausible; moreover, 7 patients including our own had additional aneurysms at other sites, while 3 had only in other locations. Only 3 patients had no intracranial aneurysms.

Each end of the fenestration is a potential place for aneurysm formation. Histologic specimens of a fenestrated basilar artery revealed thinning and even disappearance of the muscularis layer at both ends of the fenestration (26) pointing to structural wall weaknesses in fenestrated segments that can lead to aneurysm formation. Furthermore, the observation that in 20 cases (87%) intracranial aneurysms were present, may reflect a lack of vascular immaturity and vulnerability to developing aneurysms at the fenestration site or elsewhere. Fenestrations can be mistaken for aneurysms as reported by Weil et al. (27).

Treatment of supraclinoid ICA fenestration related aneurysms was performed for 19 aneurysms (Table 1): 9 open surgery, 8 endovascular, either by coiling alone, balloon/stent assistance or flow-diversion, and in one case with 2 aneurysms treatment was performed by a combined surgical and endovascular approach. No procedural complications were encountered during endovascular treatment; interestingly one limb of the fenestration was sacrificed in two cases (14, 16) with no clinical consequences. Moreover, in two cases (21, 22) flow-diverters were successfully deployed inside one limb of the fenestration without intra- or postprocedural complications. Flow-diverting stents can be a promising therapeutic option, if recanalization of fenestration-associated aneurysms occurs. Despite this, one should be cautious because perforators can emerge from either limb as noticed by Onoda et al. (11) during craniotomy. Due to the “friendly” dome-to-neck ratio (i.e., ~2), we were able to treat our patient by simple coiling, without procedural complications and an uneventful postoperative course.

To our knowledge, we presented the 9^th^ case of a supraclinoid ICA fenestration associated aneurysm treated by endovascular coil occlusion, providing further evidence that in such cases this therapeutic option is safe and effective, without significant procedural complications.

## Conclusion

Supraclinoid ICA fenestrations are rare anatomical variations. There appears to be a causative relationship between them and associated aneurysms suggesting the need for careful analysis of imaging and angiographic studies related to these patients. Endovascular treatment of supraclinoid ICA fenestration-related aneurysms is feasible and safe, with the notable concern of perforators originating from the limbs.

## Data availability statement

The original contributions presented in the study are included in the article/supplementary material, further inquiries can be directed to the corresponding author.

## Ethics statement

The studies involving human participants were reviewed and approved by Ethics Committee of Târgu-Mureṣ Emergency County Hospital, Romania. The patients/participants provided their written informed consent to participate in this study. Written informed consent was obtained from the individual(s) for the publication of any potentially identifiable images or data included in this article.

## Author contributions

Conceptualization, writing—original draft preparation and methodology: RF and LM. Software and data curation: EA. Validation: All authors. Formal analysis, investigation, and resources: ER, AM, and CC. Writing—review and editing and visualization: RF. All authors have read and agreed to the published version of the manuscript.

## Conflict of interest

The authors declare that the research was conducted in the absence of any commercial or financial relationships that could be construed as a potential conflict of interest.

## Publisher's note

All claims expressed in this article are solely those of the authors and do not necessarily represent those of their affiliated organizations, or those of the publisher, the editors and the reviewers. Any product that may be evaluated in this article, or claim that may be made by its manufacturer, is not guaranteed or endorsed by the publisher.
